# 1-(2-Benzoyl-1-phenyl­eth­yl)-4-[(2-hy­droxy-1-naphth­yl)methyl­idene­amino]-3-methyl-1*H*-1,2,4-triazole-5(4*H*)-thione

**DOI:** 10.1107/S1600536810052979

**Published:** 2011-01-08

**Authors:** Wei Wang, Yan Gao, Zuo-bing Xiao, Hong-guo Yao, Jing-jing Zhang

**Affiliations:** a School of Perfume and Aroma Technology, Shanghai Institute of Technology, Shanghai 200235, People’s Republic of China; b School of Chemical Engineering, University of Science and Technology LiaoNing, Anshan 114051, People’s Republic of China

## Abstract

In the title compound, C_29_H_24_N_4_O_2_S, intra­molecular O—H⋯N hydrogen bonding influences the mol­ecular conform­ation; the naphthol system and triazole ring form a dihedral angle of 3.88 (7)°. In the crystal, π–π inter­actions between the five- and six-membered rings of neighbouring mol­ecules [centroid–centroid distances = 3.541 (3) and 3.711 (3) Å] consolidate the crystal packing.

## Related literature

For details of the pharmacological properties of Mannich bases, see: Joshi *et al.* (2004 ▶); Ferlin *et al.* (2002 ▶); Holla *et al.* (2003 ▶). For their application in the polymer indusry, see: Negm *et al.* (2005 ▶). For standard bond lengths, see: Allen *et al.* (1987 ▶).
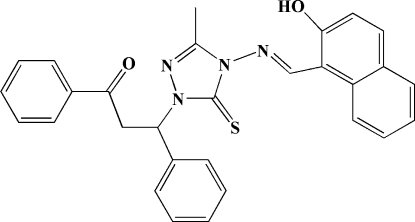


## Experimental

### 

#### Crystal data

C_29_H_24_N_4_O_2_S
*M* *_r_* = 492.58
Monoclinic, 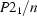

*a* = 7.8192 (16) Å
*b* = 20.248 (4) Å
*c* = 15.360 (3) Å
β = 94.69 (3)°
*V* = 2423.7 (8) Å^3^
*Z* = 4
Mo *K*α radiation
μ = 0.17 mm^−1^
*T* = 153 K
0.18 × 0.16 × 0.10 mm

#### Data collection

Rigaku Saturn CCD area-detector diffractometer
Absorption correction: multi-scan (*CrystalClear*; Rigaku/MSC, 2005 ▶) *T*_min_ = 0.970, *T*_max_ = 0.983
23596 measured reflections
5746 independent reflections
4491 reflections with *I* > 2σ(*I*)
*R*_int_ = 0.048

#### Refinement

*R*[*F* ^2^ > 2σ(*F* ^2^)] = 0.054
*wR*(*F* ^2^) = 0.127
*S* = 0.96
5746 reflections
331 parameters
3 restraints
H atoms treated by a mixture of independent and constrained refinement
Δρ_max_ = 0.31 e Å^−3^
Δρ_min_ = −0.30 e Å^−3^

### 

Data collection: *CrystalClear* (Rigaku/MSC, 2005 ▶); cell refinement: *CrystalClear*; data reduction: *CrystalClear*; program(s) used to solve structure: *SHELXS97* (Sheldrick, 2008 ▶); program(s) used to refine structure: *SHELXL97* (Sheldrick, 2008 ▶); molecular graphics: *SHELXTL* (Sheldrick, 2008 ▶); software used to prepare material for publication: *SHELXTL*.

## Supplementary Material

Crystal structure: contains datablocks global, I. DOI: 10.1107/S1600536810052979/cv5020sup1.cif

Structure factors: contains datablocks I. DOI: 10.1107/S1600536810052979/cv5020Isup2.hkl

Additional supplementary materials:  crystallographic information; 3D view; checkCIF report

## Figures and Tables

**Table 1:** Hydrogen-bond geometry (Å, °)

*D*—H⋯*A*	*D*—H	H⋯*A*	*D*⋯*A*	*D*—H⋯*A*
O1—H1⋯N1	0.89 (3)	1.83 (3)	2.610 (2)	145 (2)

**Fig. 1.**
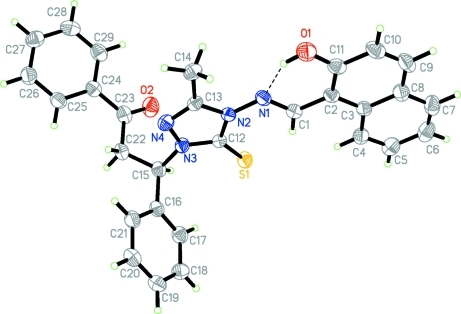
View of the molecule of (I) showing the atom-labelling scheme. Displacement ellipsoids are drawn at the 60% probability level. Dashed line denotes the hydrogen bond.

## supplementary crystallographic information

### Comment

Mannich bases have been reported as potential biological agents and received considerable attention due to their pharmacological properties - antitubercular (Joshi *et al.*, 2004), vasorelaxing (Ferlin *et al.*, 2002), anticancer (Holla *et al.*, 2003), and due to their applications in the polymer industry as paints and surface active agents (Negm *et al.*, 2005). Herein we report the synthesis and crystal structure of the title compound, (I).

In (I) (Fig. 1), the bond lengths and angles are found to have normal values (Allen *et al.*, 1987). An intramolecular O—H···N hydrogen bond results in the formation of a planar (r.m.s. deviation = 0.0262 (2) Å) six-membered ring (Table 2) and influences the molecular conformation - the naphthol system and triazole ring form a dihedral angle of 3.88 (7)°. Two phenyl rings are located on the two sides of the triazole ring. They form a dihedral angle of 34.2 (3)°.

In the crystal structure, π-π interactions (Table 1) between the five- and six-mebered rings from the neighbouring molecules consolidate the crystal packing.

### Experimental

The title compound was synthesized by the reaction of the chalcone (2.0 mmol) with its corresponding Schiff base, which was in turn obtained by refluxing 4-amino-1-methyl-4*H*-1,2,4-triazole-5-thiol (2.0 mmol), 2-hydroxynaphthalene-1 -carbaldehyde (2.0 mmol) in ethanol. A mixture of Schiff base and chalcone in ethanol was stirring for 24 h.The reaction progress was monitored *via* TLC. The resulting precipitate was filtered off, washed with cold ethanol, dried and purified to give the target product as colorless solid in 84% yield. Crystals of (I) suitable for single-crystal X-ray analysis were grown by slow evaporation of a solution in chloroform-ethanol (1:1).

### Refinement

The H atom attached to O atom was located on a difference map and isotropically refined. Other H atoms were positioned geometrically (C—H = 0.95–0.99 Å)  and refined as riding, with *U*_iso_(H) = 1.2-1.5 *U*_eq_ of the parent atom.

### Crystal data

**Table:**

C_29_H_24_N_4_O_2_S	*F*(000) = 1032
*M**_r_* = 492.58	*D*_x_ = 1.350 Mg m^−^^3^
Monoclinic, *P*2_1_/*n*	Mo *K*α radiation, λ = 0.71073 Å
Hall symbol: -P 2yn	Cell parameters from 6032 reflections
*a* = 7.8192 (16) Å	θ = 1.7–27.9°
*b* = 20.248 (4) Å	µ = 0.17 mm^−^^1^
*c* = 15.360 (3) Å	*T* = 153 K
β = 94.69 (3)°	Prism, colourless
*V* = 2423.7 (8) Å^3^	0.18 × 0.16 × 0.10 mm
*Z* = 4	

### Data collection

**Table:**

Rigaku Saturn CCD area-detector diffractometer	5746 independent reflections
Radiation source: rotating anode	4491 reflections with *I* > 2σ(*I*)
multilayer	*R*_int_ = 0.048
Detector resolution: 7.31 pixels mm^-1^	θ_max_ = 27.9°, θ_min_ = 1.7°
φ and ω scans	*h* = −10→10
Absorption correction: multi-scan (*CrystalClear*; Rigaku/MSC, 2005)	*k* = −26→26
*T*_min_ = 0.970, *T*_max_ = 0.983	*l* = −17→20
23596 measured reflections	

### Refinement

**Table:**

Refinement on *F*^2^	Secondary atom site location: difference Fourier map
Least-squares matrix: full	Hydrogen site location: inferred from neighbouring sites
*R*[*F*^2^ > 2σ(*F*^2^)] = 0.054	H atoms treated by a mixture of independent and constrained refinement
*wR*(*F*^2^) = 0.127	*w* = 1/[σ^2^(*F*_o_^2^) + (0.06*P*)^2^ + 1.1394*P*] where *P* = (*F*_o_^2^ + 2*F*_c_^2^)/3
*S* = 0.96	(Δ/σ)_max_ = 0.001
5746 reflections	Δρ_max_ = 0.31 e Å^−^^3^
331 parameters	Δρ_min_ = −0.30 e Å^−^^3^
3 restraints	Extinction correction: *SHELXL97* (Sheldrick, 2008), Fc^*^=kFc[1+0.001xFc^2^λ^3^/sin(2θ)]^-1/4^
Primary atom site location: structure-invariant direct methods	Extinction coefficient: 0.0261 (17)

### Special details

**Table:**

Geometry. All e.s.d.'s (except the e.s.d. in the dihedral angle between two l.s. planes) are estimated using the full covariance matrix. The cell e.s.d.'s are taken into account individually in the estimation of e.s.d.'s in distances, angles and torsion angles; correlations between e.s.d.'s in cell parameters are only used when they are defined by crystal symmetry. An approximate (isotropic) treatment of cell e.s.d.'s is used for estimating e.s.d.'s involving l.s. planes.
Refinement. Refinement of *F*^2^ against ALL reflections. The weighted *R*-factor *wR* and goodness of fit *S* are based on *F*^2^, conventional *R*-factors *R* are based on *F*, with *F* set to zero for negative *F*^2^. The threshold expression of *F*^2^ > σ(*F*^2^) is used only for calculating *R*-factors(gt) *etc*. and is not relevant to the choice of reflections for refinement. *R*-factors based on *F*^2^ are statistically about twice as large as those based on *F*, and *R*- factors based on ALL data will be even larger.

### Fractional atomic coordinates and isotropic or equivalent isotropic displacement parameters (Å^2^)

**Table:**

	*x*	*y*	*z*	*U*_iso_*/*U*_eq_	
S1	0.28857 (5)	0.20831 (2)	−0.04504 (3)	0.02895 (15)	
O1	0.77192 (16)	−0.01503 (7)	−0.07589 (10)	0.0358 (3)	
H1	0.760 (3)	0.0246 (14)	−0.0519 (16)	0.071 (9)*	
O2	0.64680 (17)	0.36195 (7)	−0.03561 (8)	0.0396 (4)	
N1	0.61510 (18)	0.09480 (7)	−0.04258 (9)	0.0249 (3)	
N2	0.60991 (17)	0.15359 (7)	0.00352 (9)	0.0210 (3)	
N3	0.55703 (17)	0.24391 (7)	0.06788 (9)	0.0227 (3)	
N4	0.72369 (17)	0.22717 (7)	0.09686 (9)	0.0249 (3)	
C1	0.4776 (2)	0.07206 (9)	−0.08296 (11)	0.0246 (4)	
H1A	0.3737	0.0963	−0.0827	0.030*	
C2	0.4795 (2)	0.00970 (8)	−0.12901 (11)	0.0237 (4)	
C3	0.3266 (2)	−0.01202 (9)	−0.17983 (11)	0.0252 (4)	
C4	0.1714 (2)	0.02452 (9)	−0.18680 (12)	0.0288 (4)	
H4	0.1656	0.0655	−0.1570	0.035*	
C5	0.0298 (3)	0.00162 (10)	−0.23590 (12)	0.0351 (5)	
H5	−0.0730	0.0269	−0.2393	0.042*	
C6	0.0336 (3)	−0.05847 (11)	−0.28135 (12)	0.0401 (5)	
H6	−0.0655	−0.0736	−0.3155	0.048*	
C7	0.1806 (3)	−0.09494 (10)	−0.27597 (12)	0.0374 (5)	
H7	0.1831	−0.1357	−0.3065	0.045*	
C8	0.3299 (2)	−0.07301 (9)	−0.22563 (11)	0.0310 (4)	
C9	0.4830 (3)	−0.11108 (9)	−0.21919 (12)	0.0350 (5)	
H9	0.4860	−0.1515	−0.2505	0.042*	
C10	0.6248 (3)	−0.09121 (9)	−0.16955 (13)	0.0340 (5)	
H10	0.7247	−0.1181	−0.1654	0.041*	
C11	0.6248 (2)	−0.03069 (9)	−0.12387 (11)	0.0280 (4)	
C12	0.4817 (2)	0.20104 (8)	0.00885 (11)	0.0218 (4)	
C13	0.7525 (2)	0.17231 (9)	0.05596 (11)	0.0228 (4)	
C14	0.9162 (2)	0.13542 (9)	0.06260 (12)	0.0287 (4)	
H14A	0.9996	0.1582	0.1032	0.043*	
H14B	0.9607	0.1329	0.0049	0.043*	
H14C	0.8968	0.0907	0.0841	0.043*	
C15	0.4795 (2)	0.30482 (8)	0.09933 (11)	0.0250 (4)	
H15	0.3971	0.3220	0.0515	0.030*	
C16	0.3790 (2)	0.29017 (8)	0.17815 (11)	0.0246 (4)	
C17	0.2021 (2)	0.28203 (9)	0.16602 (12)	0.0304 (4)	
H17	0.1450	0.2880	0.1096	0.037*	
C18	0.1083 (2)	0.26531 (10)	0.23552 (13)	0.0343 (5)	
H18	−0.0125	0.2598	0.2264	0.041*	
C19	0.1902 (2)	0.25662 (10)	0.31833 (13)	0.0350 (5)	
H19	0.1264	0.2444	0.3658	0.042*	
C20	0.3654 (2)	0.26592 (10)	0.33109 (12)	0.0329 (4)	
H20	0.4217	0.2607	0.3878	0.039*	
C21	0.4598 (2)	0.28280 (9)	0.26172 (12)	0.0297 (4)	
H21	0.5802	0.2894	0.2713	0.036*	
C22	0.6197 (2)	0.35606 (9)	0.11750 (11)	0.0282 (4)	
H22A	0.5679	0.3971	0.1386	0.034*	
H22B	0.7029	0.3396	0.1645	0.034*	
C23	0.7142 (2)	0.37200 (9)	0.03791 (12)	0.0292 (4)	
C24	0.8901 (2)	0.39992 (8)	0.05155 (11)	0.0267 (4)	
C25	0.9478 (2)	0.42988 (9)	0.13012 (12)	0.0313 (4)	
H25	0.8764	0.4305	0.1773	0.038*	
C26	1.1086 (2)	0.45870 (10)	0.13988 (12)	0.0343 (5)	
H26	1.1462	0.4801	0.1931	0.041*	
C27	1.2149 (2)	0.45643 (10)	0.07221 (13)	0.0339 (4)	
H27	1.3257	0.4760	0.0790	0.041*	
C28	1.1596 (2)	0.42573 (10)	−0.00498 (13)	0.0356 (5)	
H28	1.2336	0.4235	−0.0509	0.043*	
C29	0.9982 (2)	0.39815 (9)	−0.01643 (12)	0.0332 (4)	
H29	0.9606	0.3780	−0.0705	0.040*	

### Atomic displacement parameters (Å^2^)

**Table:**

	*U*^11^	*U*^22^	*U*^33^	*U*^12^	*U*^13^	*U*^23^
S1	0.0224 (3)	0.0312 (3)	0.0323 (3)	0.00355 (18)	−0.00375 (19)	−0.00608 (19)
O1	0.0287 (8)	0.0362 (8)	0.0423 (8)	0.0057 (6)	0.0020 (6)	−0.0040 (7)
O2	0.0469 (8)	0.0435 (9)	0.0273 (8)	−0.0095 (7)	−0.0036 (6)	0.0027 (6)
N1	0.0272 (8)	0.0238 (8)	0.0241 (8)	0.0011 (6)	0.0048 (6)	−0.0021 (6)
N2	0.0205 (7)	0.0225 (7)	0.0203 (7)	0.0015 (6)	0.0026 (6)	−0.0005 (5)
N3	0.0197 (7)	0.0238 (7)	0.0241 (8)	0.0012 (6)	−0.0013 (6)	−0.0012 (6)
N4	0.0200 (7)	0.0269 (8)	0.0273 (8)	0.0017 (6)	−0.0018 (6)	0.0015 (6)
C1	0.0253 (9)	0.0251 (9)	0.0238 (9)	0.0022 (7)	0.0037 (7)	0.0000 (7)
C2	0.0283 (9)	0.0220 (8)	0.0216 (9)	0.0004 (7)	0.0065 (7)	0.0022 (7)
C3	0.0316 (10)	0.0252 (9)	0.0194 (9)	−0.0038 (7)	0.0069 (7)	0.0012 (7)
C4	0.0320 (10)	0.0292 (9)	0.0253 (9)	−0.0031 (8)	0.0025 (8)	0.0015 (7)
C5	0.0347 (11)	0.0413 (11)	0.0284 (10)	−0.0054 (9)	−0.0017 (8)	0.0047 (8)
C6	0.0463 (13)	0.0472 (13)	0.0260 (10)	−0.0173 (10)	−0.0031 (9)	0.0020 (9)
C7	0.0547 (13)	0.0327 (11)	0.0252 (10)	−0.0127 (10)	0.0052 (9)	−0.0018 (8)
C8	0.0441 (12)	0.0273 (9)	0.0228 (9)	−0.0063 (8)	0.0094 (8)	0.0015 (7)
C9	0.0551 (13)	0.0219 (9)	0.0298 (10)	0.0003 (9)	0.0151 (9)	−0.0016 (7)
C10	0.0409 (11)	0.0252 (9)	0.0374 (11)	0.0073 (8)	0.0121 (9)	0.0006 (8)
C11	0.0316 (10)	0.0273 (9)	0.0260 (9)	0.0021 (8)	0.0088 (8)	0.0034 (7)
C12	0.0206 (9)	0.0230 (8)	0.0222 (9)	−0.0010 (7)	0.0037 (7)	−0.0003 (7)
C13	0.0207 (8)	0.0256 (9)	0.0223 (9)	−0.0023 (7)	0.0019 (7)	0.0033 (7)
C14	0.0220 (9)	0.0307 (10)	0.0332 (10)	0.0021 (8)	0.0010 (7)	0.0031 (8)
C15	0.0257 (9)	0.0224 (9)	0.0265 (9)	0.0034 (7)	0.0005 (7)	−0.0020 (7)
C16	0.0276 (9)	0.0201 (8)	0.0260 (9)	0.0029 (7)	0.0012 (7)	−0.0030 (7)
C17	0.0287 (10)	0.0328 (10)	0.0291 (10)	0.0024 (8)	−0.0015 (8)	−0.0029 (8)
C18	0.0266 (10)	0.0385 (11)	0.0383 (11)	0.0007 (8)	0.0059 (8)	−0.0059 (9)
C19	0.0383 (11)	0.0366 (11)	0.0312 (11)	−0.0004 (9)	0.0103 (9)	−0.0065 (8)
C20	0.0379 (11)	0.0353 (11)	0.0252 (10)	0.0014 (9)	0.0016 (8)	−0.0040 (8)
C21	0.0287 (10)	0.0304 (10)	0.0297 (10)	0.0011 (8)	0.0003 (8)	−0.0050 (8)
C22	0.0321 (10)	0.0252 (9)	0.0270 (10)	−0.0006 (8)	0.0005 (8)	−0.0038 (7)
C23	0.0381 (11)	0.0226 (9)	0.0263 (10)	0.0009 (8)	−0.0002 (8)	0.0015 (7)
C24	0.0332 (10)	0.0199 (8)	0.0271 (9)	0.0015 (7)	0.0038 (8)	0.0015 (7)
C25	0.0342 (10)	0.0316 (10)	0.0284 (10)	0.0008 (8)	0.0055 (8)	−0.0037 (8)
C26	0.0354 (11)	0.0331 (10)	0.0342 (11)	−0.0013 (9)	0.0008 (8)	−0.0050 (8)
C27	0.0309 (10)	0.0299 (10)	0.0417 (11)	0.0006 (8)	0.0064 (8)	0.0041 (8)
C28	0.0388 (11)	0.0369 (11)	0.0328 (11)	0.0050 (9)	0.0129 (9)	0.0045 (9)
C29	0.0430 (11)	0.0313 (10)	0.0252 (10)	0.0011 (9)	0.0032 (8)	0.0006 (8)

### Geometric parameters (Å, °)

**Table:**

S1—C12	1.6679 (18)	C14—H14A	0.9800
O1—C11	1.352 (2)	C14—H14B	0.9800
O1—H1	0.89 (3)	C14—H14C	0.9800
O2—C23	1.223 (2)	C15—C22	1.518 (2)
N1—C1	1.282 (2)	C15—C16	1.526 (2)
N1—N2	1.3873 (19)	C15—H15	1.0000
N2—C13	1.374 (2)	C16—C17	1.390 (2)
N2—C12	1.396 (2)	C16—C21	1.392 (3)
N3—C12	1.355 (2)	C17—C18	1.386 (3)
N3—N4	1.3842 (19)	C17—H17	0.9500
N3—C15	1.473 (2)	C18—C19	1.388 (3)
N4—C13	1.305 (2)	C18—H18	0.9500
C1—C2	1.448 (2)	C19—C20	1.380 (3)
C1—H1A	0.9500	C19—H19	0.9500
C2—C11	1.397 (2)	C20—C21	1.388 (2)
C2—C3	1.442 (2)	C20—H20	0.9500
C3—C4	1.418 (2)	C21—H21	0.9500
C3—C8	1.423 (2)	C22—C23	1.514 (2)
C4—C5	1.369 (3)	C22—H22A	0.9900
C4—H4	0.9500	C22—H22B	0.9900
C5—C6	1.404 (3)	C23—C24	1.486 (2)
C5—H5	0.9500	C24—C25	1.393 (2)
C6—C7	1.363 (3)	C24—C29	1.397 (2)
C6—H6	0.9500	C25—C26	1.383 (3)
C7—C8	1.417 (3)	C25—H25	0.9500
C7—H7	0.9500	C26—C27	1.384 (3)
C8—C9	1.421 (3)	C26—H26	0.9500
C9—C10	1.354 (3)	C27—C28	1.377 (3)
C9—H9	0.9500	C27—H27	0.9500
C10—C11	1.412 (2)	C28—C29	1.378 (3)
C10—H10	0.9500	C28—H28	0.9500
C13—C14	1.478 (2)	C29—H29	0.9500
			
Cg1···Cg1^i^	3.541 (3)	Cg2···Cg3^ii^	3.711 (3)
			
C11—O1—H1	109.0 (16)	H14B—C14—H14C	109.5
C1—N1—N2	119.78 (14)	N3—C15—C22	108.79 (13)
C13—N2—N1	118.65 (13)	N3—C15—C16	110.50 (14)
C13—N2—C12	109.15 (14)	C22—C15—C16	113.46 (14)
N1—N2—C12	132.19 (14)	N3—C15—H15	108.0
C12—N3—N4	113.88 (14)	C22—C15—H15	108.0
C12—N3—C15	125.99 (14)	C16—C15—H15	108.0
N4—N3—C15	120.09 (13)	C17—C16—C21	118.86 (16)
C13—N4—N3	104.41 (13)	C17—C16—C15	119.23 (15)
N1—C1—C2	120.53 (16)	C21—C16—C15	121.88 (16)
N1—C1—H1A	119.7	C18—C17—C16	120.61 (17)
C2—C1—H1A	119.7	C18—C17—H17	119.7
C11—C2—C3	119.12 (16)	C16—C17—H17	119.7
C11—C2—C1	121.57 (16)	C17—C18—C19	120.22 (18)
C3—C2—C1	119.30 (15)	C17—C18—H18	119.9
C4—C3—C8	117.74 (16)	C19—C18—H18	119.9
C4—C3—C2	123.25 (16)	C20—C19—C18	119.41 (18)
C8—C3—C2	119.01 (16)	C20—C19—H19	120.3
C5—C4—C3	120.92 (18)	C18—C19—H19	120.3
C5—C4—H4	119.5	C19—C20—C21	120.57 (18)
C3—C4—H4	119.5	C19—C20—H20	119.7
C4—C5—C6	121.22 (19)	C21—C20—H20	119.7
C4—C5—H5	119.4	C20—C21—C16	120.30 (17)
C6—C5—H5	119.4	C20—C21—H21	119.8
C7—C6—C5	119.41 (19)	C16—C21—H21	119.8
C7—C6—H6	120.3	C23—C22—C15	112.96 (14)
C5—C6—H6	120.3	C23—C22—H22A	109.0
C6—C7—C8	121.07 (19)	C15—C22—H22A	109.0
C6—C7—H7	119.5	C23—C22—H22B	109.0
C8—C7—H7	119.5	C15—C22—H22B	109.0
C7—C8—C9	121.31 (18)	H22A—C22—H22B	107.8
C7—C8—C3	119.64 (18)	O2—C23—C24	121.14 (16)
C9—C8—C3	119.05 (17)	O2—C23—C22	120.53 (16)
C10—C9—C8	121.64 (18)	C24—C23—C22	118.33 (15)
C10—C9—H9	119.2	C25—C24—C29	119.08 (17)
C8—C9—H9	119.2	C25—C24—C23	121.24 (16)
C9—C10—C11	120.27 (18)	C29—C24—C23	119.65 (16)
C9—C10—H10	119.9	C26—C25—C24	120.33 (17)
C11—C10—H10	119.9	C26—C25—H25	119.8
O1—C11—C2	123.05 (17)	C24—C25—H25	119.8
O1—C11—C10	116.08 (16)	C25—C26—C27	120.08 (18)
C2—C11—C10	120.87 (17)	C25—C26—H26	120.0
N3—C12—N2	101.83 (14)	C27—C26—H26	120.0
N3—C12—S1	127.02 (13)	C28—C27—C26	119.75 (18)
N2—C12—S1	131.07 (13)	C28—C27—H27	120.1
N4—C13—N2	110.67 (15)	C26—C27—H27	120.1
N4—C13—C14	125.43 (16)	C27—C28—C29	120.85 (17)
N2—C13—C14	123.88 (16)	C27—C28—H28	119.6
C13—C14—H14A	109.5	C29—C28—H28	119.6
C13—C14—H14B	109.5	C28—C29—C24	119.88 (17)
H14A—C14—H14B	109.5	C28—C29—H29	120.1
C13—C14—H14C	109.5	C24—C29—H29	120.1
H14A—C14—H14C	109.5		
			
C1—N1—N2—C13	171.14 (15)	N3—N4—C13—N2	−0.84 (18)
C1—N1—N2—C12	−8.5 (3)	N3—N4—C13—C14	177.86 (15)
C12—N3—N4—C13	−0.75 (19)	N1—N2—C13—N4	−177.60 (14)
C15—N3—N4—C13	−178.93 (14)	C12—N2—C13—N4	2.09 (19)
N2—N1—C1—C2	−177.96 (14)	N1—N2—C13—C14	3.7 (2)
N1—C1—C2—C11	6.5 (3)	C12—N2—C13—C14	−176.63 (15)
N1—C1—C2—C3	−174.89 (15)	C12—N3—C15—C22	−146.60 (16)
C11—C2—C3—C4	178.02 (15)	N4—N3—C15—C22	31.3 (2)
C1—C2—C3—C4	−0.6 (2)	C12—N3—C15—C16	88.2 (2)
C11—C2—C3—C8	−2.0 (2)	N4—N3—C15—C16	−93.84 (17)
C1—C2—C3—C8	179.36 (15)	N3—C15—C16—C17	−98.27 (18)
C8—C3—C4—C5	0.1 (2)	C22—C15—C16—C17	139.25 (17)
C2—C3—C4—C5	−179.94 (16)	N3—C15—C16—C21	79.84 (19)
C3—C4—C5—C6	−0.4 (3)	C22—C15—C16—C21	−42.6 (2)
C4—C5—C6—C7	0.5 (3)	C21—C16—C17—C18	−1.5 (3)
C5—C6—C7—C8	−0.3 (3)	C15—C16—C17—C18	176.63 (17)
C6—C7—C8—C9	179.46 (17)	C16—C17—C18—C19	0.1 (3)
C6—C7—C8—C3	0.0 (3)	C17—C18—C19—C20	1.1 (3)
C4—C3—C8—C7	0.1 (2)	C18—C19—C20—C21	−1.0 (3)
C2—C3—C8—C7	−179.90 (15)	C19—C20—C21—C16	−0.4 (3)
C4—C3—C8—C9	−179.36 (15)	C17—C16—C21—C20	1.7 (3)
C2—C3—C8—C9	0.7 (2)	C15—C16—C21—C20	−176.46 (16)
C7—C8—C9—C10	−178.40 (17)	N3—C15—C22—C23	58.68 (19)
C3—C8—C9—C10	1.0 (3)	C16—C15—C22—C23	−177.89 (14)
C8—C9—C10—C11	−1.3 (3)	C15—C22—C23—O2	22.8 (2)
C3—C2—C11—O1	−178.19 (15)	C15—C22—C23—C24	−157.10 (15)
C1—C2—C11—O1	0.4 (3)	O2—C23—C24—C25	160.25 (18)
C3—C2—C11—C10	1.7 (2)	C22—C23—C24—C25	−19.9 (2)
C1—C2—C11—C10	−179.67 (16)	O2—C23—C24—C29	−17.6 (3)
C9—C10—C11—O1	179.87 (16)	C22—C23—C24—C29	162.27 (17)
C9—C10—C11—C2	−0.1 (3)	C29—C24—C25—C26	1.4 (3)
N4—N3—C12—N2	1.92 (18)	C23—C24—C25—C26	−176.53 (17)
C15—N3—C12—N2	179.96 (14)	C24—C25—C26—C27	−1.6 (3)
N4—N3—C12—S1	−175.26 (12)	C25—C26—C27—C28	0.4 (3)
C15—N3—C12—S1	2.8 (2)	C26—C27—C28—C29	1.2 (3)
C13—N2—C12—N3	−2.33 (17)	C27—C28—C29—C24	−1.4 (3)
N1—N2—C12—N3	177.30 (15)	C25—C24—C29—C28	0.1 (3)
C13—N2—C12—S1	174.67 (13)	C23—C24—C29—C28	178.06 (17)
N1—N2—C12—S1	−5.7 (3)		

Symmetry codes: (i) −*x*+3/2, *y*+3/2, −*z*−1/2; (ii) −*x*+1/2, *y*+1/2, −*z*−1/2.

### Hydrogen-bond geometry (Å, °)

**Table:**

*D*—H···*A*	*D*—H	H···*A*	*D*···*A*	*D*—H···*A*
O1—H1···N1	0.89 (3)	1.83 (3)	2.610 (2)	145 (2)
